# Combinatorial hypofractionated radiotherapy and pembrolizumab in anaplastic thyroid cancer

**DOI:** 10.1530/ETJ-23-0144

**Published:** 2024-01-29

**Authors:** Janice Ser Huey Tan, Timothy Kwang Yong Tay, Enya Hui Wen Ong, Michael Fehlings, Daniel Shao-Weng Tan, Nadiah Binte Sukma, Eileen Xueqin Chen, Jen-Hwei Sng, Connie Siew Poh Yip, Kok Hing Lim, Darren Wan-Teck Lim, Narayanan Gopalakrishna Iyer, Jacqueline Siok Gek Hwang, Melvin Lee Kiang Chua, Mei-Kim Ang

**Affiliations:** 1 Division of Radiation Oncology, National Cancer Centre Singapore, Hospital Boulevard, Singapore; 2 Department of Pathology, Singapore General Hospital, Singapore; 3 ImmunoScape, 1 Scotts Road #24-10, Singapore; 4 Division of Medical Oncology, National Cancer Centre Singapore, Hospital Boulevard, Singapore; 5 Division of Surgical Oncology, National Cancer Centre Singapore, Hospital Boulevard, Singapore

**Keywords:** hypofractionated radiotherapy, immunotherapy, anaplastic thyroid cancer, QUAD-shot

## Abstract

**Objective:**

Anaplastic thyroid cancer (ATC) is an aggressive disease associated with poor outcomes and resistance to therapies. Our study aim was to evaluate the activity of a combinatorial regimen of sandwich sequencing of pembrolizumab immunotherapy and hypofractionated radiotherapy (RT).

**Methods:**

In this case series, patients with ATC received hypofractionated RT (QUAD-shot) and intravenous pembrolizumab 200 mg every 3–4 weeks. Pembrolizumab was continued until disease progression or up till 24 months. Concurrent lenvatinib treatment was allowed. Primary endpoint was best overall response (BOR) and progression-free survival (PFS). Additionally, we performed immune profiling of circulating T cells in a responder to investigate the immune response to our combinatorial treatment.

**Results:**

At median follow-up of 32.6 months (IQR: 26.4–38.8), of a cohort of five patients, BOR was 80%; with two complete responses (CR) and two partial responses (PR). Patients who achieved CR remained disease-free at last follow-up. Median PFS was 7.6 months (IQR: 6.2–NR), and 1-year PFS and overall survival rate was 40% (95% CI: 13.7–100) for both. Treatment was well-tolerated, with mostly grade 1–2 adverse events. Immune profiling of one partial responder revealed an increase in activated CD4 and CD8 T cells post-QUAD-shot RT, which was further enhanced during the maintenance phase of pembrolizumab.

**Conclusion:**

Herein, we report a case series of five patients with ATC, with two long-term survivors who were treated with surgical debulking followed by QUAD-shot RT and pembrolizumab, possibly due to synergy of local and systemic treatments in activating anti-tumour immunogenic cytotoxicity. This regimen warrants further investigation in a larger cohort of patients.

## Introduction

Anaplastic thyroid cancers (ATCs) are rare aggressive tumours, comprising only 1–2% of all thyroid cancers worldwide, and are typically associated with high mortality rates and a median overall survival (OS) of 5–12 months (1, 2, 3). ATCs typically develop from differentiated tumours as a result of one or more de-differentiating steps, in particular, loss of function of TP53 protein, since approximately 50% of patients have either a prior or coexistent diagnosis of differentiated thyroid cancer (DTC). Unlike DTC, ATCs cannot concentrate iodine, do not express the thyroid-stimulating hormone (TSH) receptor and do not produce thyroglobulin. Therefore, ATCs are typically resistant to radioactive iodine (RAI), thyroxine suppression and other therapies.

Recent evaluations of the molecular landscape of ATC have begun to elucidate molecular drivers that are associated with ATC tumourigenesis, which has identified opportunities for targeted therapies (4, 5, 6). Between 20% and 50% of ATCs harbour activating *BRAF V600E* mutations. A recent phase 2 trial evaluating dabrafenib and trametinib in 16 patients with *BRAF V600E*-mutated ATC reported an objective overall response rate (ORR) of 69%, 12-month progression-free survival (PFS) and OS rates of 79% and 80%, respectively (7). *NTRK* fusions have been also detected in 5–25% of all thyroid cancers, and in a recent phase 1–2 basket trial of an *NTRK* inhibitor – larotrectinib, the ORR amongst 28 patients with thyroid cancer (including seven patients with ATC) was 75%, with a duration of response ranging from 1.9 to 41.0 months; median PFS was not reached (8, 9). *RET* fusions are another class of mutations that have been detected in 5–10% of thyroid cancers (excluding medullary thyroid cancers), and a durable response was achieved in a patient with ATC who received selpercatinib, a selective *RET* inhibitor, as part of the LIBRETTO-001 phase 1/2 pan-tumour study (10, 11).

Nonetheless, despite ongoing efforts to characterise the molecular landscape and evaluate novel targeted therapeutics for ATC, treatment options remain limited for the majority of ATC patients. Whilst multitargeted tyrosine kinase inhibitors like lenvatinib and sorafenib have been established as standards of care in advanced DTC (12, 13), lenvatinib did not yield similar activity when used as monotherapy in ATC (14, 15), suggesting that perhaps a combination regimen is needed. In this respect, a retrospective study of a combination of pembrolizumab and lenvatinib reported the best ORR of 66% and a median PFS of 16.5 months amongst six patients with ATC (16, 17). The subsequent phase II ATLEP study demonstrated a comparable ORR with this combination (18, 19). Immune checkpoint inhibitors (ICIs) such as pembrolizumab target the programmed death-1/-ligand 1 (PD-1/PD-L1) axis and have demonstrated activity in head and neck squamous cell carcinoma (HNSCC), where the seminal KEYNOTE-048 trial established the role of pembrolizumab in the first-line treatment of recurrent/metastatic HNSCC (20). Interestingly, ATC has a high immune cell-infiltrated tumour microenvironment that is enriched by tumour-associated macrophages predominantly and high PD-L1 expression (21, 22, 23). Thus, ICIs targeting the CTLA4 and PD1–PDL1 axis have been investigated in advanced thyroid cancers and have shown modest ORR amongst ATC patients (24). Separately, radiotherapy (RT) could synergise with ICI through local and systemic immunostimulation by the release of tumour neoantigens and upregulation of MHC class I molecules, activation of dendritic cells, and enhanced cross-presentation of antigens, as well as increasing the density of tumour-infiltrating lymphocytes (25). Conversely, RT could also lead to immunosuppression through the upregulation of PD-1/-L1, which lends itself to the concept of adjuvant ICI post-RT that proved to be successful in the definitive treatment of non-small cell lung cancer (NSCLC) (26).

Here, we report a case series of five patients with ATC who underwent a combinatorial regimen of sandwich sequencing of pembrolizumab and accelerated hypofractionated RT (QUAD-shot). We demonstrate that such a combination was able to achieve a high ORR, resulting in two long-term survivors. Additionally, we performed longitudinal deep profiling of the systemic immune response in one responder, and demonstrated the synergy between ICI and QUAD-shot RT in activating cytotoxic and helper T cells.

## Materials and methods

### Patient selection

Consecutive patients with newly diagnosed ATC at a single institution with ECOG ≤2, adequate organ function, no active autoimmune disease and contraindications to immunotherapy were included. Diagnosis and pathology of all patients were confirmed at the institution’s multidisciplinary tumour board. Patients provided written consent for the release of their clinical information for this case series under the Singapore Health Services Centralised Institutional Review Board (CIRB ref. no.: 2019/2177).

### Treatment procedures

Pembrolizumab was administered intravenously at a fixed dose of 200 mg every 3–4 weeks. Treatment was continued until progression or up to 24 months in the absence of progression. In the event of ≥grade 3 immune-related adverse events (irAEs), treatment was discontinued. In two patients, lenvatinib was given concurrently with pembrolizumab, based on the phase II ATLEP study (18), with the dose left to the individual physician’s discretion.

QUAD-shot RT regimen was delivered in between cycles of pembrolizumab, commencing 10–14 days after the last cycle of pembrolizumab. This regimen consisted of bi-daily fractions of 3.5 Gy, which were delivered at intervals of >6 h for 2 consecutive days, resulting in a total of 14 Gy over four fractions. This may be repeated every 3–4 weeks for a total of up to four cycles (cumulative dose of 56 Gy), depending on the tumour control status and/or toxicities. All treatments were delivered using a megavoltage linear accelerator with 6 MV photons. Treatment planning was performed using volumetric modulated arc therapy with image guidance by cone-beam CT (CBCT) scans. For patients with two or more treatment cycles, adaptive radiotherapy (ART) was performed to adjust to anatomic changes and tumour response.

### PD-L1 immunohistochemistry and tumour molecular and immune profiling

PD-L1 status was determined through immunohistochemistry (antibody clone 22C3; Dako or SP263; Ventana) in tumour tissue specimens obtained at initial diagnosis or surgery prior to treatment. The tumour was then assessed by a pathologist for the tumour proportion score (TPS) and/or the combined positive score (CPS). Molecular testing was performed by next-generation sequencing (NGS) or the *BRAF V600E* real-time PCR assay. The NGS assay (Somatic Solid Tumour Panel, or SSTP) was a customised amplicon-based assay that interrogates mutational hotspots and targeted regions in 26 genes including *BRAF*, *KRAS*, *NRAS*, *PIK3CA* and *TP53*. The real-time PCR assay is a TaqMan probe-based allele-specific real-time PCR assay that was modified from the protocol by Benlloch *et al.* (27), and tests only for the *BRAF c.1799T>A, p.V600E* mutation. No screen for fusions was performed in this case series.

For systemic immune profiling, we applied mass cytometry (cytometry by time of flight, CyTOF) to deeply characterise CD8 and CD4 T cells *ex vivo,* as described previously (28). Details of the PD-L1 immunohistochemistry, NGS, real-time PCR assay and immune profiling methods are provided in the Supplementary Appendix (see section on supplementary materials given at the end of this article).

### Statistical considerations

The primary endpoints of this case series were to determine BOR and PFS resulting from a combination of pembrolizumab and QUAD-shot RT, based on the Response Evaluation Criteria in Solid Tumour (RECIST) version 1.1 (29). Other endpoints included OS and treatment-related adverse events (TRAEs). PFS was defined as the time from starting treatment to the occurrence of progression/death, whilst OS was defined as the time from starting treatment to the occurrence of death. Kaplan–Meier curves were used to analyse OS and PFS. TRAEs were reported and graded according to National Cancer Institute (NCI) Common Terminology Criteria for Adverse Events (CTCAE version 5). Descriptive statistics were used to summarise patients’ characteristics. Statistical significance was set at a threshold of *P*-value of <0.05. Plots were made using R version 4.0.2.

## Results

### Patient cohort

Between March 2019 and September 2022, five patients with newly diagnosed ATC were treated with a combination of QUAD-shot RT to the primary thyroid tumour or tumour bed, concurrent with 3–4 weekly pembrolizumab infusions.

Baseline demographics and disease characteristics are summarised in Table 1. None of the patients received prior RT or RAI. Notably, patients 4 and 5 received oral lenvatinib at a dose ranging from 8 to 14 mg daily concurrent with pembrolizumab and RT. Patient 5 was initially misdiagnosed as stage 4 NSCLC and had received one cycle of paclitaxel–carboplatin–pembrolizumab before being switched to pembrolizumab–lenvatinib combined with RT. Whilst patient 3 had undergone a total thyroidectomy, she had measurable residual disease at the time of the study.

**Table 1:** Baseline demographics and disease characteristics.

Characteristics	Patients				
	1	2	3	4	5
Age (years)	51	74	64	73	56
Gender	F	F	F	F	M
ECOG status	1	1	0	2	1
Surgery type	TT	ND	TT	Tracheostomy	ND
Resection status	R2	ND	R2	Thyroid mass *in situ*	ND
Disease site	Thyroid bed, oesophagus, distant LN, lung and muscle	Primary *in situ*, cervical LN	Thyroid bed, Oesophagus	Thyroid, thyro-oesophageal groove and oesophagus	Primary *in situ*, lung, mediastinal LN
PD-L1					
TPS	75%	90%	<5%	2%	95%
CPS	80		70	10	
Molecular profiling	NRAS p.Q61K	BRAF p.V600E	Negative for BRAF p.V600E	KRAS p.L19F, TP53 p.M169*	NRAS p.Q61K, PIK3CA p.H1047R, TP53 p.R248W

CPS, combined positive score; ECOG status, Eastern Cooperative Oncology Group performance status; LN, lymph nodes; NA, not done; PD-L1, programmed death-ligand 1; R1, R1 resection with removal of all macroscopic disease but microscopic margins positive for tumour; R2, R2 resection with gross residual disease; TT, total thyroidectomy; ; ; TPS, tumour proportion score.

### Outcome of treatment and adverse effects

**Figure 1:**
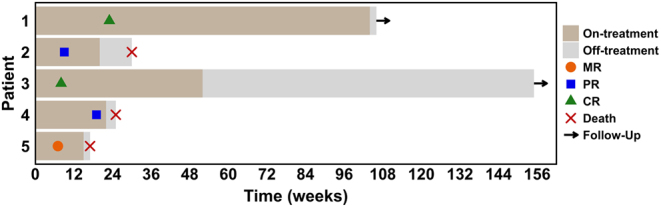
Swimmer’s plot showing the treatment duration, response and progression-free survival in the five patients. MR, mixed response; PR, partial response; CR, complete response.

**Figure 2:**
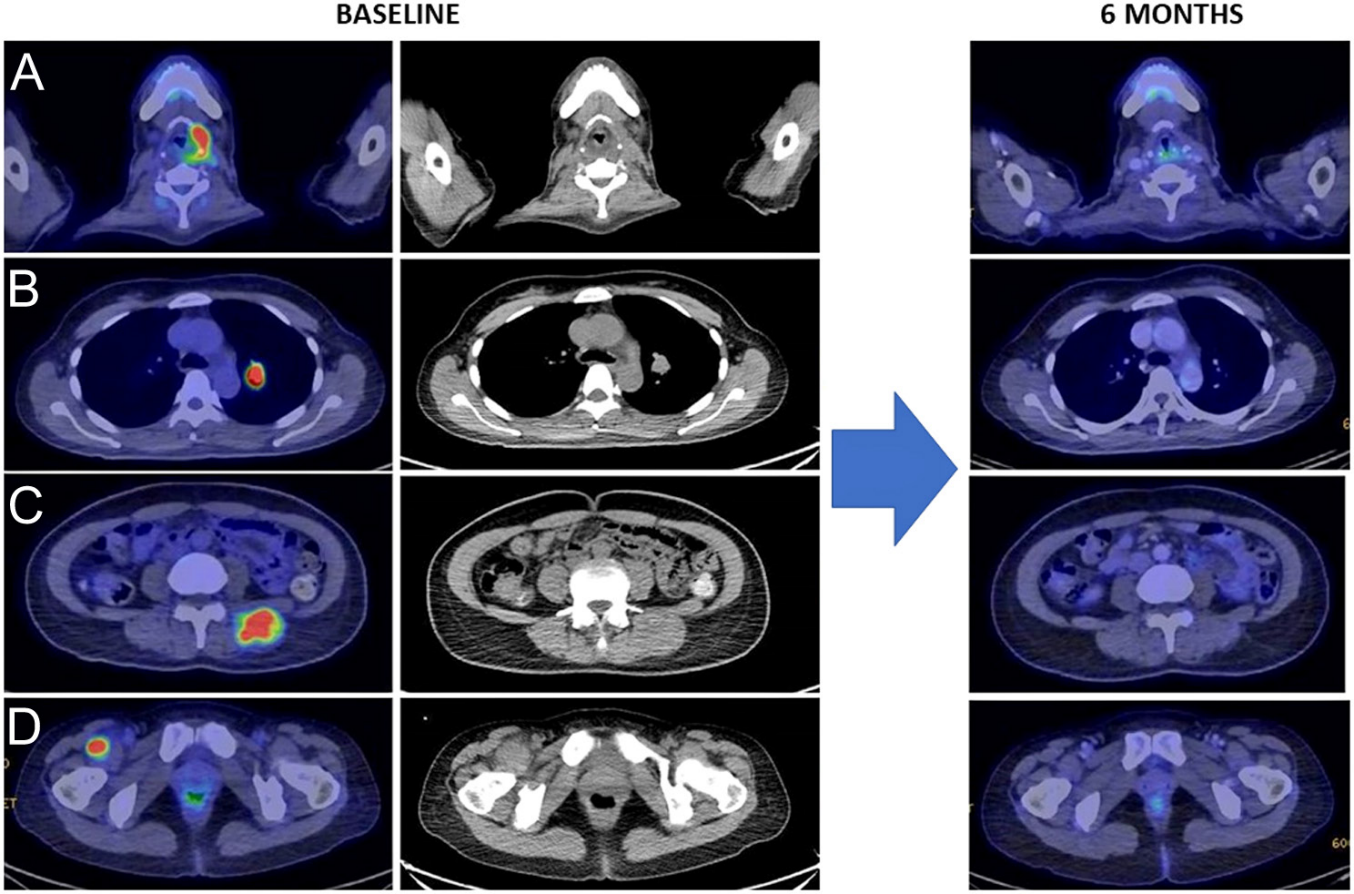
Serial FDG-PET/CT scans at baseline and at the 6-month timepoint from patient 1 showing complete response at both local and distant metastatic sites at 6 months after treatment initiation. A. Thyroid bed. B. Left hilar lymph node. C and D. Muscle.

As of October 2022, with a median follow-up of 32.6 (IQR: 26.4–38.8) months, no patients were on active treatment. We observed an ORR of 80%, with two complete responses (CR) and two partial responses (PR) (Table 2 and Fig. 1). Of the complete responders, patients 1 and 3 obtained a CR within 6 months (Fig. 2) and 2 months, respectively, and remained disease-free at their last follow-up despite both having stopped treatment. Patient 3 elected to stop treatment after 12 months of maintenance pembrolizumab due to the COVID-19 pandemic, whilst patient 1 completed 24 months of pembrolizumab. For the partial responders, patients 2 and 4 achieved PR at 3 and 5 months, respectively. Both patients succumbed to death subsequently due to severe pneumonia at 8 and 5 months during the maintenance phase of pembrolizumab (Table 2 and Fig. 1), immune-mediated pneumonitis was excluded in both patients. Patient 2 had significant dysphagia and was on naso-gastric tube feeding; her chest X-ray and CT scan showed infective changes. Patient 4 similarly had infective change on X-ray, and sputum culture was positive for *Klebsiella pneumoniae*.

**Table 2:** Treatment details, adverse effects and outcomes of treatment.

Details	Patients				
	1	2	3	4	5
No. of QUAD-shot cycles	2	4	2	4	1
Duration of PEMBRO (weeks)	104	20	52	22	15
Other therapy	None^a^	None	None	Lenvatinib for 3 months	Lenvatinib for 3 months
Adverse effects	RT: xerostomia G1; ICI: dry skin G1	Laryngeal oedema G3; dysphagia G2; anorexia G1; breathlessness G1; giddiness G1; fatigue G2	Nil	RT: cough G1; Lenvatinib: anorexia G1; fatigue G2	Nil
BOR, RECIST 1.1	CR at 6 months	PR at 3 months	CR at 2 months	PR at 5 months	MR at 2 months (PD brain, bone)
PFS (months)	NR	8	NR	6	2
OS (months)	NR	8	NR	6	4
Current status	CR, alive	Passed away from pneumonia	CR, alive	Passed away from pneumonia	Passed away from cancer

BOR, best overall response based on RECIST 1.1; CR, complete response; ICI, immune checkpoint inhibitor; MR, mixed response; PR, partial response; PD, progressive disease; NR, not reached.

^a^No other treatment was added to pembrolizumab and radiotherapy.

**Figure 3:**
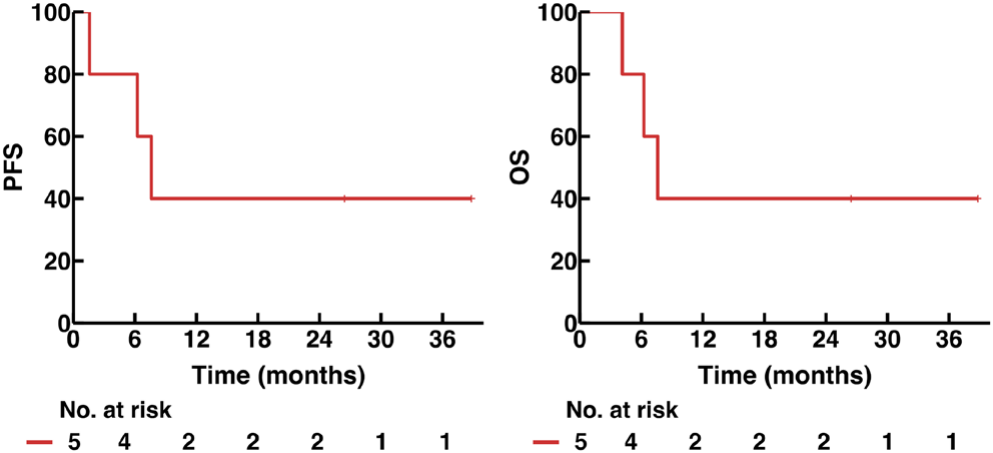
Kaplan–Meier curves showing progression-free survival (PFS) and overall survival (OS) in the five patients.

For the non-responder (patient 5), this patient demonstrated a mixed response after two cycles of pembrolizumab and one cycle of QUAD-shot RT and lenvatinib, with improvement in the primary thyroid mass, lung and lymph node metastases, but with progression in the bones and brain. As there was derived clinical benefit, coupled with limited treatment options, the patient was maintained on pembrolizumab, but progressed at 4 months with bowel obstruction due to tumour spread. For this patient, the cause of death was disease progression of ATC. Overall, median PFS for our cohort was 7.6 (IQR: 6.2-NR) months, and 1-year PFS and OS rates were 40% (95% CI: 13.7–100) for both (Fig. 3).

The treatment was well tolerated, with patients reporting mostly grade 1–2 AE. Notably, patient 2 developed severe laryngeal oedema after one cycle of QUAD-shot RT, which improved after treatment with steroids. Although she completed four cycles of QUAD-shot RT, she experienced side effects such as dysphagia, anorexia, breathlessness, and giddiness during maintenance pembrolizumab. Patient 4 developed anorexia and fatigue due to lenvatinib, which resolved upon discontinuation of lenvatinib after 3 months.

### Molecular profiling and biomarker analysis

PD-L1 immunohistochemistry was performed for all patients, which revealed high PD-L1 TPS/ CPS of ≥50 for all, except for patient 4 (CPS of 10). Targeted testing for the *BRAF V600E* mutation was performed on patients 2 and 3, whilst NGS was done for patients 1, 4 and 5. Patient 2 was found to have a *BRAF V600E* mutation and was offered the combination of dabrafenib and trametinib, which the patient declined due to cost and opted for the study treatment instead. Amongst all patients who underwent NGS testing, alterations in the RAS pathway were the most common (Table 1).

### Longitudinal deep immune profiling

**Figure 4:**
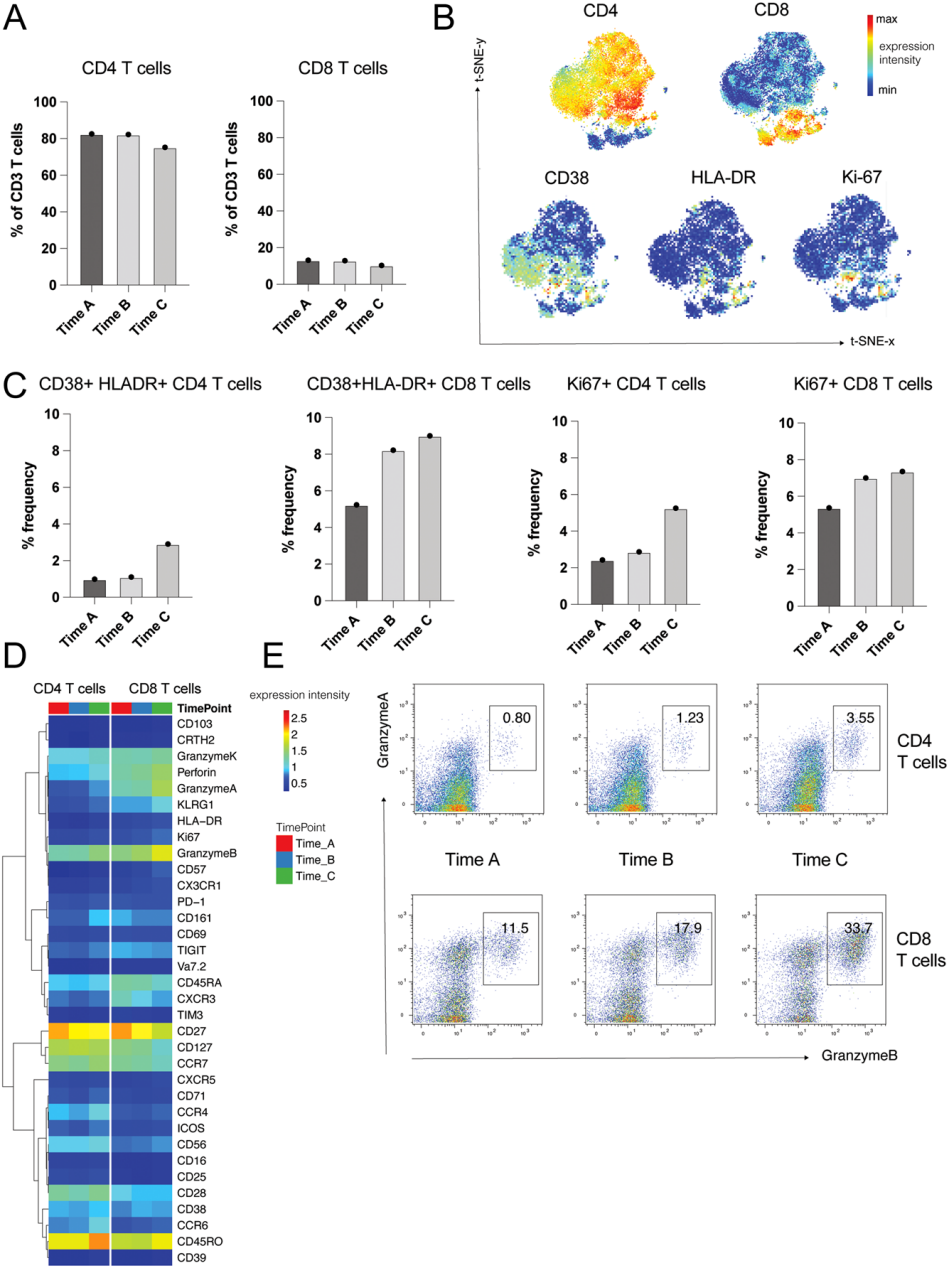
A. Frequencies of CD4 and CD8 T cells amongst the overall CD3 T-cell population across the timepoints for patient 4. B. Uniform manifold approximation and projection (UMAP) dimensionality reduction technique visualising immune cell subsets with individual plots showing the relative position of cells expressing CD4, CD8, CD38, HLA-DR and Ki67. C. Frequencies of activated T cells across the three timepoints; pre-ICI and RT (time A), 6 months (time B) and 12 months post-ICI and radiotherapy (RT; time C). D. Expression of phenotypic markers of CD4 and CD8 T cells across the timepoints in our extreme responder. We observed persistent high expression of CD27 and CD45RO, and increased expression of granzyme B post-RT. E. Increased proportion of granzyme B+ CD4 and CD8 T cells following RT.

To investigate the immune response underpinning the response to our combinatorial treatment, we performed immune profiling of circulating T cells in a responder (patient 4 (Fig. 4)). Consistent with our previous observations (28), we observed an increase in activated (CD38^+^HLA-DR^+^ and Ki67^+^) CD4 and CD8 T cells post-QUAD-shot RT, which was further enhanced during the maintenance phase of pembrolizumab (Fig. 4C). Next, gene expression analysis of immune phenotypic markers in CD4 and CD8 T-cell populations revealed a persistent expression of CD27 and CD45RO across the timepoints, suggesting that these immune-stimulatory cells were present at baseline in this patient (Fig. 4D). Finally, we observed an increase in the proportion of granzyme B+ CD4 and CD8 T cells post-QUAD-shot RT that was sustained during the maintenance phase of pembrolizumab in this patient (Fig. 4E).

## Discussion

Here, we report a case series of five patients with ATC who underwent a combinatorial regimen involving sandwich sequencing of ICI therapy with pembrolizumab and accelerated hypofractionated RT (QUAD-shot). We demonstrate that such a combination yielded objective responses in four of five patients, and impressively, maximal responses were observed within 6 months of starting treatment. Additionally, we recorded two long-term survivors in our cohort, which is a remarkable outcome in a disease notorious for its dismal prognosis. Furthermore, both of these patients did not harbour *BRAF V600E* mutations, suggesting this treatment has activity in non-*BRAF* patients. On the other hand, both these patients had undergone upfront thyroidectomy. Thus, it is plausible that in these survivors, surgery played a role in reducing tumour bulk, which then favoured the ability of QUAD-shot RT and pembrolizumab in targeting the remnant disease through immunogenic cell death. RT- and ICI-related toxicities were mostly mild (G1), and none of the patients discontinued treatment due to TRAEs. One patient experienced severe (G3) laryngeal oedema shortly after one cycle of pembrolizumab, which may be attributable to the combination treatment, although this patient had her primary tumour *in situ* and declined an elective tracheostomy procedure. Nevertheless, the patient’s symptoms resolved with repeated courses of steroids, and she eventually managed to complete four cycles of QUAD-shot RT. Of the three deaths that occurred, two were not related to disease progression or treatment-related toxicities but instead were due to pneumonia. Patients who developed complications such as pneumonia and laryngeal oedema had their primary tumour *in situ*. Thus, surgery can offer good local control in the management of ATC and should be considered where feasible.

The outcomes of our combinatorial treatment are comparable to other treatment regimens in ATC. In particular, in a recent phase 2 trial, 71 patients with ATC (one-third of whom harboured distant metastases) were randomized to weekly paclitaxel, daily pazopanib and concurrent radiotherapy or paclitaxel and radiotherapy alone. Overall survival in the control arm was 7.3 months, and the response rate was 33.3% (30).

Taken together, these findings support the efficacy of this combinatorial regimen in achieving good local and distant disease control whilst remaining largely safe and well tolerated. Additionally, we performed longitudinal deep profiling of the systemic immune response in one responder and demonstrated that this combination was able to induce a shift in the systemic immune response of this patient. We observed a substantial increase in the frequencies of activated CD4 and CD8 T cells post-QUAD-shot RT, which was further enhanced during the maintenance phase of pembrolizumab. These effector T cells also displayed significant upregulation of activating immune markers such as CD27, CD45RO and markers of cytotoxicity (granzymes A and B).

The molecular landscape of ATC is characterised by a high tumour mutational burden, an immunologically ‘hot’ tumour microenvironment with high expression of various inhibitory immune-checkpoint mediators, including PD-L1, and increased neoangiogenesis (VEGFR/FGFR signalling) (6, 31), all of which provide a strong mechanistic rationale for combining ICI with anti-angiogenic agents. Lenvatinib is an oral multi-kinase inhibitor (VEGFR1-3, PDGFR, FGFR1-4, RET and c-KIT) that has demonstrated synergistic activity with pembrolizumab in pre-clinical studies (32) through its anti-angiogenic effects and ability to enhance immune cell infiltration into the tumour, thus augmenting the ICI-induced immunogenic response. In the phase 2 ATLEP trial, the combination of pembrolizumab with lenvatinib was associated with a BOR within 2 years of 51.9% PR and 44.4% stable disease amongst 27 patients with ATC (19). In our case series, two patients received the combination of pembrolizumab and lenvatinib, albeit at a lower lenvatinib dose. However, we did not observe an associated benefit from the addition of lenvatinib in this small patient cohort, as good responses were seen even with pembrolizumab and RT alone.

As described earlier, RT has been shown to synergise with ICI because of both local and systemic RT-induced immunomodulation. This balance of immunostimulatory and immunoinhibitory signals renders RT alone insufficient to induce an anti-tumour immune response, thus it would be logical to combine RT with ICI to maximally exploit the anti-tumour immune response by RT. This combinatorial strategy has proven to be successful in the landmark PACIFIC trial, where there was a significant OS benefit of adjuvant durvalumab (anti-PD-L1 antibody) after definitive chemoradiation in stage III NSCLC (26). In contrast, several combination ICI-RT trials performed in HNSCC have not yielded a similar success (33, 34, 35). These conflicting results could be explained by several mechanisms, including (i) the target volumes that involve comprehensive irradiation of the lymph node stations, which could be detrimental in triggering an anti-tumour immunogenic response (36); (ii) sequencing of ICI before RT, which may increase the radiosensitivity of CD8 T cells leading to depletion of these tumour-specific effector T cells, and dampening of the systemic antitumour response (37); and (iii) deploying the optimal RT dose-fractionation, since hypofractionated RT has been shown to yield significantly better local and distal (abscopal) tumour control compared with conventional 1.8–2 Gy fractionation (38). Taken together, we hypothesise that the high ORR seen in our series of patients with ATC could be explained by the sandwich sequencing of ICI/RT, which catered for sufficient rest periods between RT courses to facilitate recovery of lymphocytes, and localised RT to the primary tumour bed.

Some limitations of this study deserve mention. First, this was a small case series of five patients, which limits the ability to generalise our findings to the general population of patients with ATC. Secondly, systemic treatment was heterogeneous with variable durations of pembrolizumab, and some patients receiving pembrolizumab and lenvatinib at different doses, and with one patient receiving prior chemotherapy. Thus, it was not possible to determine the relative contribution of lenvatinib vs pembrolizumab. Thirdly, one patient with *BRAF V600E* was offered this study treatment in lieu of targeted therapy. Lastly, patients 2 and 3 did not have measurable disease outside of the RT field, hence the contribution of immunotherapy versus RT cannot be distinguished fully. The optimal sequencing of immunotherapy and RT in these patients is also debatable, given the importance of RT for local control. However, the presence of durable responses in these patients who did not receive lenvatinib (patients 1 and 3), as well as the prolonged duration of local and distant control in these patients, suggest that in some patients, the combination of pembrolizumab and QUAD-shot RT following surgical debulking may be sufficient. Thus, the promising results and durable responses warrant a larger scale systematic investigation, since such a combination of RT and pembrolizumab may prove to be more cost-effective compared with other drug combinations and may be considered particularly in patients who do not harbour targetable alterations. Nevertheless, whilst adverse events in our series were mostly low grade and manageable, further careful evaluation of this regimen is required, given the possibility for serious life-threatening adverse events to occur.

Looking ahead, in the context of evolving therapies and increasing knowledge of different molecular subtypes of ATC, it will be important to explore further therapeutic options in conjunction with in-depth molecular profiling, as well as correlative biomarker analyses to discern the optimal treatment choice and sequence of therapies in order to achieve rapid durable disease control. Personalised approach to immunotherapy-based combination therapies is currently ongoing where patients with ATC and poorly DTC are stratified into four treatment cohorts based on mutation status (Clinicaltrials.gov, NCT03181100), and will receive atezoliumab in combination with vemurafenib plus cobimetinib, or cobimetinib alone, or bevacizumab or paclitaxel chemotherapy. Initial results from this study showed promising results with median OS of 18.23 months and 1-year OS of 67% (39). Interestingly there is an ongoing pilot clinical trial in advanced recurrent/metastatic HNSCC (NCT04454489) investigating pembrolizumab in combination with QUAD-shot RT to gross disease prior to cycles 3, 7 and 12. The primary endpoint of this study is ORR, with correlative analyses to evaluate the possible biomarkers of response.

## Conclusion

In this article, we report a case series of five patients with ATC, with two long-term survivors who were treated with surgical debulking followed by a sandwich regimen of pembrolizumab and QUAD-shot RT. Treatment was well tolerated, with no patient stopping treatment due to toxicity. Immune profiling validated the synergy between pembrolizumab and QUAD-shot RT in activating circulating T cells, which could account for treatment efficacy. Our results warrant further investigation of this combinatorial regimen in a larger cohort of patients who may be amendable to upfront surgery.

## Supplementary materials

Supplementary Material

## Declaration of interest

The authors declare that there is no conflict of interest that could be perceived as prejudicing the impartiality of the study reported.

## Funding

Melvin L. K. Chua is supported by the National Medical Research Councilhttp://dx.doi.org/10.13039/501100000265 Singapore Clinician Scientist Award (NMRC/CSA-INV/0027/2018, CSAINV20nov-0021), the Duke-NUS Oncology Academic Program, Goh Foundation Proton Research Programme, NCCS Cancer Fund and the Kua Hong Pak Head and Neck Cancer Research Programme.

## Authors’ disclosure

All named authors meet the International Committee of Medical Journal Editors (ICMJE) criteria for authorship for this article, take responsibility for the integrity of the work as a whole and have given their approval for this version to be published. We certify that the manuscript has not been under consideration for publication elsewhere, nor has it been submitted or accepted in another publication in any form.

## Author contribution statement

JSHT performed investigations, formal analysis, data curation and writing. TKYT developed methodology, performed investigations and writing. EHWO performed formal analysis and writing. MF developed methodology, performed investigation, formal analysis and writing. DSWT conceived the study, developed methodology, performed investigations and writing. NBS developed methodology, performed investigation and writing. EXC developed methodology, performed investigation and writing. JHS developed methodology, performed investigation and writing. CSPY developed methodology, performed investigation and writing. KHL developed methodology, performed investigation and writing. DWTL performed investigation and writing. NGI developed methodology, performed investigation and writing. JSGH developed methodology, performed investigation and writing. MLKC conceived the study, developed methodology, performed investigations, formal analysis, data curation and writing. MKA conceived the study, developed methodology, performed investigation, formal analysis, data curation and writing.
