# Supplementary material for: Combinatorial hypofractionated radiotherapy and pembrolizumab in anaplastic thyroid cancer
Source: Eur Thyroid J. 2024 Jan 29;13(1):e230144. doi: 10.1530/ETJ-23-0144 (PMC10895326; doi:10.1530/ETJ-23-0144)
Supplement: Supplementary Material [file supplementary_material.pdf]

Supplementary Appendix

Methodology

Immunohistochemistry

PD-L1 testing

PD-L1 status was determined by immunohistochemistry (antibody clone 22C3; Dako or SP263; Ventana) in tumor tissue specimens obtained at initial diagnosis or surgery prior to treatment. For PD-L1 SP263 clone, paraffin sections of formalin fixed tissue were stained using the Roche Ventana ready-to-use antibody (reference number: 741-4905). The stain was performed in Ventana Benchmark Ultra system using Ventana Optiview detection kit. Antigen retrieval was performed using CC1 buffer for 64mins. For PD-L1 22C3 clone, paraffin sections of formalin fixed tissue were stained using a closed system FDA-approved PHARMDX kit by DAKO. The paraffin sections went through antigen retrieval by EnVision Flex in PT LINK and the stain was done in DAKO AUTOSTAINER LINK 48.

Tumour mutational analysis using next generation sequencing

DNA extraction and library preparation

The NGS assay (Somatic Solid Tumour Panel or SSTP) is an amplicon based NGS assay that interrogates mutational hotspots and targeted regions in 26 genes (Supplementary Table 1 below), using an input of only 10 - 20 ng of input DNA. Genomic DNA was extracted from formalin-fixed paraffin-embedded (FFPE) tumour tissue using the Qiagen AllPrep® DNA / RNA FFPE Kit (QIAGEN GmbH, Hilden, Germany) according to the manufacturer’s recommendations. This kit allows for the

simultaneous extraction and purification of both total RNA and genomic DNA from the same input FFPE tissue.

Libraries were then prepared from tumour DNA using the Ion Torrent Ampliseq 2.0 kit (ThermoFisher Scientific) and the different sub-panels (Ion Ampliseq™ Colon and Lung Cancer Panel V2 and Ampliseq customised SSTPv1.5) according to the manufacturer's instructions. The resulting amplicons were partially digested, phosphorylated and barcoded using the Ion Xpress™ Barcode Adaptor 1 – 96 kit (ThermoFisher Scientific). These were subsequently purified and the quality and concentration of the libraries were determined using Qubit™ dsDNA HS Assay Kits with the Qubit® 2.0 Fluorometer (ThermoFisher Scientific).

Sequencing on the Ion PGM platform

Libraries were then pooled for sequencing on the Ion PGM 318 v2 chip. Pooled libraries were clonally amplified on Ion Spheres (ThermoFisher Scientific). The Ion PGM Hi-Q View OT2 kit and the Ion OneTouch™ 2 System (ThermoFisher Scientific) were utilised for the emulsion PCR (e-PCR) and enrichment of the template-positive Ion Sphere Particles (ISPs) that contained the clonally amplified DNA. The enriched ISPs were loaded on a PGM 318 v2 chip and sequenced on the Ion Personal Genome Machine® (PGM™) platform using the Ion PGM Hi-Q View Sequencing kit (ThermoFisher Scientific).

Data analysis for Ion PGM

Analysis of the raw sequencing data and alignment of the sequencing reads to the reference genome (Human genome build 19 of hg 19) were primarily performed by the Torrent Suite™ Variant Caller plugin software v5.2.2 for Ion PGM (ThermoFisher Scientific) and an in-house analysis pipeline v1.2.0 (for reference genome hg19).

All genomic analyses from the use of tumour tissue specimens to the analysis pipeline and database references was aimed at the detection of somatic changes. They were not designed nor validated for the interrogation of germline changes. The Integrative Genome Viewer (IGV) was used to visualise the read alignments and check the presence of the variants against the reference genome. This was also done to mitigate strand biases and sequencing errors. A minimum sequencing depth of 250X (with a target base coverage of 100%), variant allele frequency of ≥ 4% and quality score of ≥ 60 for single nucleotide variants and insertions – deletions was used.

Supplementary Table 1

**NGS Assay Somatic Solid Tumour Panel**	
**Targets**	Hotspots and target regions in 207 amplicons of 26 genes implicated in cancer

**Genes**	*AKT1, ALK, BRAF, CTNNB1, DDR2, EGFR, ERBB2, ERBB4, FBXW7, FGFR1, FGFR2, FGFR3, IDH1, IDH2, KRAS, MAP2K1, MET, NOTCH1, NRAS, PIK3CA, POLD, POLE, PTEN, SMAD4, STK11 and TP53*
**Amplicon length**	207 amplicons with an average length of 162 bp
**Primer pool**	207 primer pairs in 3 pools

Supplementary Table 2: Baseline demographics and disease characteristics.

Patient	Symptoms at Dx	Sites of disease at study baseline	Size of thyroid primary	Pre-RT gross tumour volume* (cm^3)	Concomitant pathology	Time from Dx to Rx start	Best response to study regimen
1	Enlarging neck mass, hoarse voice	Thyroid bed, oesophagus, distant LN, lung, muscle	7.5cm	108.2	Follicular thyroid cancer	6 weeks	CR
2	Left neck mass, hoarse voice	Primary in situ, cervical LN	4.7cm	97.4	None	6 weeks	PR (thyroid ↓34%; lymph node ↓35%)
3	Dysphagia, neck mass, hoarse voice	Thyroid bed, Oesophagus	Not known *(surgery done in different country)*	2	None	6 weeks	CR
4	Enlarging neck mass, hoarse voice	Primary in situ, Thyro-oesophageal groove, oesophagus	7.7cm	194.8	None	5 weeks	PR (thyroid ↓65%)
5	Neck mass, sore throat, hoarse voice	Primary in situ, lung, mediastinal LN	10.9cm	113.1	Papillary thyroid cancer	4 weeks	MR (thyroid ↓51%; lung ↓20%; lymph node ↓40%; NEW brain and bone mets)

*Dx: diagnosis; RT: radiotherapy; *based on RT planning volume; Rx: Treatment; LN: lymph nodes; CR: complete response; PR: partial response; MR: mixed response*
